# Selection and Validation of Reference Genes in *Clinacanthus nutans* Under Abiotic Stresses, MeJA Treatment, and in Different Tissues

**DOI:** 10.3390/ijms26062483

**Published:** 2025-03-11

**Authors:** Chang An, Lin Lu, Yixin Yao, Ruoyu Liu, Yan Cheng, Yanxiang Lin, Yuan Qin, Ping Zheng

**Affiliations:** 1 College of Agriculture, Guangxi University, Nanning 530004, China; ancher0928@163.com; 2 Fujian Provincial Key Laboratory of Haixia Applied Plant Systems Biology, Haixia Institute of Science and Technology, College of Life Sciences, Fujian Agriculture and Forestry University, Fuzhou 350002, China; ll15966192116@163.com (L.L.); liuruoyu13@mails.ucas.ac.cn (R.L.); chengyan1220@hotmail.com (Y.C.); 3 Macau Centre for Research and Development in Chinese Medicine, State Key Laboratory of Quality Research in Chinese Medicine, Institute of Chinese Medical Sciences, University of Macau, Macao, China; y13592925434@126.com; 4 College of Pharmacy, Fujian University of Traditional Chinese Medicine, Fuzhou 350122, China; linyanxiang@fjtcm.edu.cn

**Keywords:** *Clinacanthus nutans*, quantitative real-time PCR, reference genes, plant stress response, molecular biology techniques

## Abstract

*Clinacanthus nutans* is a valuable traditional medicinal plant that contains enriched active compounds such as triterpenoids and flavonoids. Understanding the accuulation process of these secondary metabolites in *C. nutans* requires exploring gene expression regulation under abiotic stresses and hormonal stimuli. qRT-PCR is a powerful method for gene expression analysis, with the selection of suitable reference genes being paramount. However, reports on stably expressed reference genes in *C. nutans* and even across the entire family Acanthaceae are limited. In this study, we evaluated the expression stability of 12 candidate reference genes (*CnUBQ, CnRPL, CnRPS, CnPTB1, CnTIP41, CnACT, CnUBC, CnGAPDH, Cn18S, CnCYP, CnEF1α, and CnTUB*) in *C. nutans* across different tissues and under abiotic stresses and MeJA treatment using three programs (geNorm, NormFinder, and BestKeeper). The integrated ranking results indicated that *CnUBC*, *CnRPL*, and *CnCYP* were the most stably expressed genes across different tissues. Under abiotic stress conditions, *CnUBC*, *CnRPL*, and *CnEF1α* were the most stable, while under MeJA treatment, *CnRPL*, *CnEF1α*, and *CnGAPDH* exhibited the highest stability. Additionally, *CnRPL*, *CnUBC*, and *CnEF1α* were the most stable reference genes across all tested samples, whereas *CnGAPDH* was the least stable. *CnRPL*, consistently ranking among the top three most stable genes, may therefore serve as an ideal reference gene for qRT-PCR analysis in *C. nutans*. To further validate the selected reference genes, we assessed the expression of two key biosynthetic genes, *CnPAL* and *CnHMGR*. The results confirmed that using the most stable reference genes yielded expression patterns consistent with biological expectations, while using unstable reference genes led to significant deviations. These findings offer valuable insights for accurately quantifying target genes via qRT-PCR in *C. nutans*, facilitating investigations into the mechanisms underlying active compound accumulation.

## 1. Introduction

*Clinacanthus nutans*, commonly known as Sabah snake grass, is a medicinal herb belonging to the family Acanthaceae (Lamiales) [1,2]. In China and many Southeast Asian countries (e.g., Malaysia, Indonesia, Thailand), this plant is highly valued in traditional herbal medicine for treating skin rashes, insect and snake bites, lesions caused by the herpes simplex virus, diabetes, and gout [3,4,5]. Phytochemical investigations have revealed that *C. nutans* is rich in bioactive compounds such as triterpenoids and flavonoids [6,7,8], which exhibit anti-inflammatory, antimicrobial, antioxidant, hepatoprotective, and hypoglycemic properties [9]. Studies have indicated that light intensity, temperature, precipitation, and fertilization can modulate the plant’s growth and bioactive compound production [10,11,12]. For instance, Fong reported that *C. nutans* leaves from plants grown at high altitudes and lower temperatures exhibited greater cytotoxicity against D24 melanoma cells than those from plants cultivated at lower altitudes with higher temperatures [13]. In medicinal plants, the accumulation, composition, and concentration of secondary metabolites are often subject to variations based on the developmental stage and environmental conditions [14,15]. These dynamic changes are primarily attributed to alterations in the expression of genes implicated in secondary metabolic pathways during developmental processes or under various stress conditions [16,17]. For instance, dehydration stress induces the expression of drought-responsive genes in soybean, leading to the upregulation of dehydrin/LEA genes and a subsequent increase in the accumulation of polysaccharides and saponins [18]. Cold stress can activate phenylpropanoid biosynthesis genes in buckwheat, significantly enhancing anthocyanin and proanthocyanidin levels [19]. Conversely, high temperatures upregulate *LhIFR* and *LhMYBC2* in lilies, resulting in increased flavonoid accumulation [20]. Additionally, various phytohormones play crucial roles in the biosynthesis of these secondary metabolites [21,22,23]. Methyl jasmonate (MeJA), as a chemical inducer, effectively promoted the accumulation of triterpenes in *Cyclocarya paliurus* [24]. Similarly, MeJA treatment significantly enhanced the total flavonoid content in licorice cells [25,26]. Moreover, MeJA has been shown to regulate the biosynthesis of indigo and indirubin in *S. cusia*, a species from the same family as *C. nutans*. To gain a comprehensive understanding of the regulatory mechanisms governing secondary metabolite accumulation in *C. nutans*, further investigations into gene expression modulation under abiotic stress and hormonal induction are warranted. Such insights will facilitate the elucidation of key biosynthetic pathways and provide a theoretical foundation for optimizing the medicinal applications of *C. nutans*.

Quantitative analysis of gene expression levels elucidates the complex regulatory network of plant secondary metabolite biosynthesis [27,28]. Quantitative real-time PCR (qRT-PCR) is widely used in gene expression analysis and molecular diagnostics due to its accuracy, high sensitivity, and broad applicability [29]. However, the accuracy of qRT-PCR results can be influenced by factors such as RNA quality, primer specificity, and amplification efficiency [30]. Therefore, it is often necessary to introduce suitable reference genes with relatively stable expression to normalize differences between samples, thereby normalizing the expression levels of target genes [31,32,33]. In plants, commonly used reference genes for qRT-PCR include various housekeeping genes, such as tubulin (*TUB*), actin (*ACT*), 18S ribosomal RNA (*18S*), translation elongation factor 1-α (*EF1α*), glyceraldehyde-3-phosphate dehydrogenase (*GAPDH*), and ubiquitin-conjugating enzyme (*UBC*) [34,35]. However, the transcription of these reference genes is not always stable across different plant species, experimental conditions, tissues, and developmental stages [36,37]. For example, *GAPDH* is considered to be one of the most stable reference genes in heading Chinese cabbage, but it is not suitable in non-heading Chinese cabbage [38]. *EF1α* is often selected as a reference gene in many plants [39,40], but it is one of the least stable genes in the leaves and roots of *Prunus* spp. rootstocks under flooding [41]. This indicates the necessity of validating the expression stability of candidate reference genes in target species before using them for normalization. Softwares such as geNorm, NormFinder, and BestKeeper have been widely used to identify the most stable reference genes in various plants, such as *Morus indica* and *Camellia oleifera* [42,43]. Therefore, it is essential to comprehensively evaluate the stably expressed genes in *C. nutans* under specific experimental conditions or in different tissues for qRT-PCR using various statistical methods.

To date, no reports have been published on reference genes for *C. nutans*. In fact, among the entire family Acanthaceae, only *Strobilanthes cusia* and *Andrographis paniculata* have undergone reference gene identification studies [28,44], which has limited gene expression analysis and related research in Acanthaceae plants. Here, we selected 12 commonly used candidate reference genes referring to the literature and recommendations from the Internal Control Gene Database (ICG) at https://doi.org/10.1093/nar/gkx875 (accessed on 12 August 2024), including *CnUBQ, CnRPL, CnRPS, CnPTB1, CnTIP41, CnACT, CnUBC, CnGAPDH, Cn18S, CnCYP, CnEF1α, and CnTUB*. The expression stability of these candidate reference genes was evaluated in different tissues of *C. nutans* under various abiotic stresses and hormonal stimulus using geNorm, NormFinder, and BestKeeper methods. Additionally, to validate the accuracy of the recommended reference genes, we used phenylalanine ammonia lyase (*PAL*) and HMG-CoA reductase (*HMGR*) as target genes. PAL is a key enzyme that catalyzes the phenylpropanoid pathway [45,46], which plays a fundamental role in the production of various compounds, such as anthocyanins, flavonoids, and phenylalanine [47]. HMGR serves as the rate-limiting enzyme within the mevalonate (MVA) pathway [48], which produces a wide variety of cyclic triterpenes [49,50,51]. This is the first systematic screening of stable reference genes for *C. nutans* under various experimental conditions. The study results provide valuable information for gene expression analysis and functional studies in *C. nutans* and other Acanthaceae plants.

## 2. Results

### 2.1. PCR Specificity and Amplification Efficiency of Candidate Reference Genes

**Figure 1:**
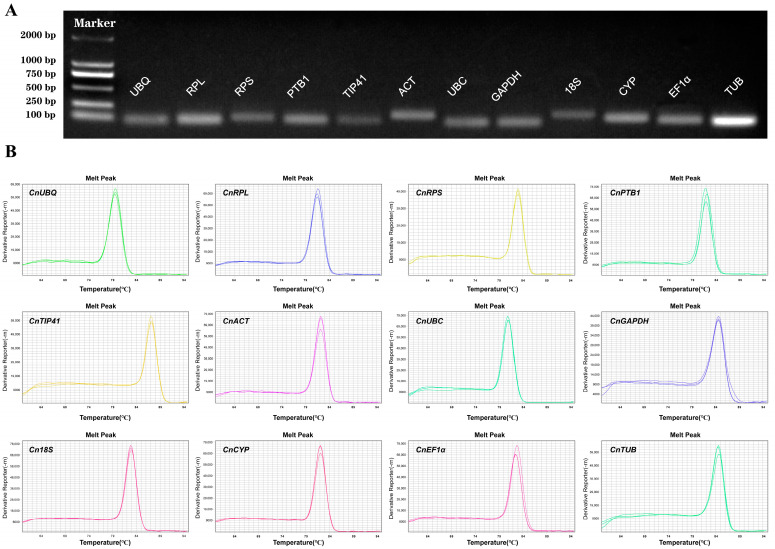
Primer specificity of 12 candidate reference genes. (**A**) Amplification length of 12 candidate reference genes determined by RT-PCR. M: DL2000 Marker. Full-length blots/gels are presented in Figure S1. (**B**) Melting curves generated for 12 candidate reference genes by qRT-PCR.

To ascertain the specificity of 12 candidate reference gene primers, RT-PCR was first performed and the products were analyzed by electrophoresis on 2% agarose gel, confirming the specificity of gene amplification for all candidate reference genes and yielding a single band of the expected length (Figure 1A, Figure S1). Melting curve analysis revealed a single peak for each primer, indicating the absence of primer dimer formation and non-specific amplification (Figure 1B). Using a 10-fold gradient dilution of the cDNA sample pool, the amplification efficiency (E) and correlation coefficient (R^2^) for all primers were calculated using the standard curve method. The amplification efficiency (E) for all primers ranged from 89.78% to 102.40%, with correlation coefficients (R^2^) ranged from 0.9925 to 0.9986 (Figure S2). The primer characteristics of all candidate reference genes are provided in Table 1.

### 2.2. Expression Profiles of Candidate Reference Genes

**Figure 2:**
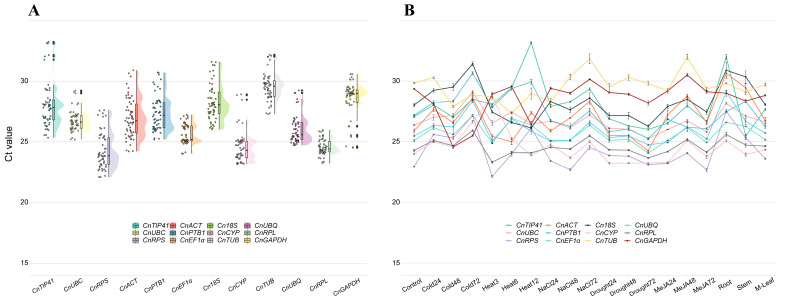
Expression profiles of 12 candidate reference genes of *C. nutans* in different tissues and under diverse stresses/MeJA treatment using qRT-PCR. (**A**) Violin and scatter plot showing the distribution of Ct values for 12 candidate reference genes. (**B**) Line chart illustrating the expression trends of Ct values under different experimental conditions. Vertical bars indicate the standard error (±SD) calculated from three biological replicates.

To assess the expression profiles of 12 candidate reference genes, the cycle threshold (Ct) values were determined in all tested samples under various abiotic stresses, MeJA stimulus, and in different tissue types (Figure 2A). The Ct value represents the quantification cycle during PCR amplification, where a lower Ct value indicates a higher expression level of the given gene. Across all samples, the Ct values of the 12 candidate reference genes ranged from 22.09 to 33.23, with mean Ct values varying between 24.27 to 29.53, and standard deviations ranging from 0.65 to 1.90 (Table S1). Among these genes, *CnRPS* was the most abundantly expressed gene with the lowest average Ct value, whereas *CnTUB* was found to have the lowest expression level with the highest average Ct value. *CnRPL* exhibited the smallest standard deviation, indicating relatively stable expression, whereas *CnTIP41* displayed the largest variability, suggesting pronounced fluctuation. Furthermore, we analyzed the expression trends for each candidate reference gene under different conditions, revealing significant fluctuations in Ct values under heat stress and across different tissues, while the Ct values remained most stable under drought stress (Figure 2B). Furthermore, under all experimental conditions, *CnRPS* exhibited significant differences when compared to the control group. With the exception of the stem tissue, *CnCYP* in other tissues and under different experimental conditions also demonstrated significant differences relative to the control group (Figure S3). Overall, the expression levels across all test samples indicated variability among the candidate reference genes, underscoring the need for further exploration to screen out more suitable reference genes.

### 2.3. Expression Stability of Candidate Reference Genes

For a comprehensive overall analysis of the optimal reference genes in *C. nutans*, we divided all test samples into four distinct subsets: “abiotic stresses” (cold, heat, NaCl, and drought), “hormonal stimulus” (MeJA), “different tissues” (root, stem, young leaf, mature leaf), and “total” (all samples tested). Then, the expression stability of the 12 candidate reference genes was analyzed and ranked based on different stability indices calculated using the geNorm, NormFinder, and BestKeeper programs (Table 2), which were commonly employed for reference gene evaluation in previous investigations [52,53].

Utilizing the geNorm algorithm, the expression stability M values of 12 candidate reference genes were calculated. A lower M value signifies more stable gene expression. Across all subsets, the M values of the 12 candidate reference genes were below the default threshold of 1.5 for classifying the reference genes as stably expressed [54]. The results demonstrated high expression stability for the candidate genes across experimental groups (Table 2). In the subset of abiotic stresses, the genes with the highest expression stability were *CnUBQ* (0.338135), *CnUBC* (0.338135), and *CnEF1α* (0.387752). In the hormonal stimulus subset, *CnRPL* (0.083542), *CnEF1α* (0.083542), and *CnGAPDH* (0.130138) were identified as the three most stable genes, all well below the 1.5 threshold. *CnRPL* (0.220736), *CnCYP* (0.220736), and *CnUBC* (0.282248) showed stable expression across different tissues of *C. nutans*. Considering all samples, *CnUBC* (0.404232) was identified as the most stable gene, followed by *CnEF1α* (0.404232) and *CnRPL* (0.464657), whereas *CnGAPDH* (1.328414) displayed the highest M value, indicating its position as the least stable gene.

**Figure 3:**
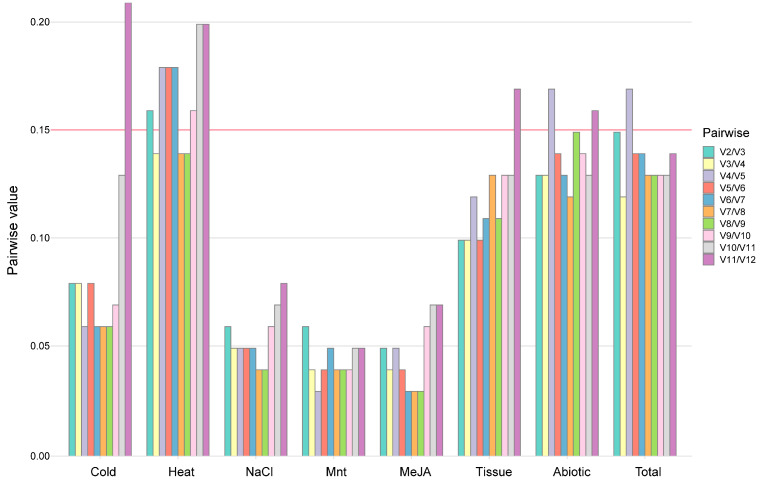
Pairwise variation of 12 candidate reference genes calculated by geNorm. “Tissue” refers to the root, stem, young leaf and mature leaf. “Abiotic” includes cold stress, heat stress, NaCl stress, and drought stress. “Hormone” indicates MeJA stimulus. “Total” represents all samples.

In accordance with the Minimum Information for Publication of Quantitative Real-time PCR Studies (MIQE) guidelines [55], we conducted pairwise variation analysis (Vn/Vn + 1) among the normalization factors of the candidate reference genes. A Vn/Vn + 1 value of less than 0.15 indicates that n reference genes are sufficient for stable normalization [56]. Given that variation analysis requires at least two samples to ensure reliable normalization, the methodological framework inherently involves a minimum of two reference genes. In most cases, normalization can be effectively achieved using only two reference genes, with the inclusion of a third gene yielding no significant improvement in data stability. As depicted in Figure 3, the pairwise variation V2/V3 values across subsets involving abiotic stresses, hormonal stimulus, and different tissues were all below 0.15, indicating that the top two combinations of reference genes were sufficiently reliable for these scenarios. However, under individual abiotic stress conditions, the V2/V3 value for heat stress was 0.16 (>0.15), suggesting that a single reference gene may not provide sufficiently representative or reproducible results under high-temperature conditions (45 °C). Consequently, when designing qPCR experiments under varying high-temperature conditions, careful consideration of the number of reference genes is essential to ensure the accuracy and reliability of gene expression analysis. Based on these findings, we propose the following combinations of reference genes as suitable for abiotic stresses (*CnUBQ* and *CnUBC*), hormonal stimuli (*CnRPL* and *CnEF1α*), and across all samples (*CnRPL* and *CnCYP*), respectively.

NormFinder calculates the stability value (SV) of each candidate reference gene using a mathematical model, assessing variability within and between groups. Similar to geNorm, a lower value means higher stability [57,58,59]. As shown in Table 2, the stability rankings of these candidate genes closely aligned with those derived from the geNorm algorithm. Specifically, except for the subset comprising different tissue samples, the top three candidate reference genes ranked by both the geNorm and NormFinder algorithms were consistent across the other three subsets, with minor variations in their stability ranking.

The BestKeeper algorithm calculates the standard deviation (SD) and coefficient of variation (CV) from the geometric mean of the Ct values of the candidate reference genes. The smaller the estimated value, the more stable the gene expression. Genes with an SD <1 are generally considered to be within an acceptable range of variation [60,61]. As shown in Table 2, under different abiotic stresses, BestKeeper analysis identified *CnRPL*, *CnUBC*, *CnEF1α*, and *CnUBQ* as the four most stable genes, consistent with the results from the geNorm and NormFinder algorithms, though the stability ranking of these genes varied slightly. However, in the hormonal stimulus and different tissue subsets, the rankings from BestKeeper analysis differed significantly from the other two software programs. For instance, *CnUBC* was identified as the most stable gene in the hormonal stimulus subset according to BestKeeper analysis, but it ranked seventh and sixth in geNorm and NormFinder analyses, respectively. In the different tissue subset, *CnTUB* and *CnGAPDH* were the two most stable genes according to BestKeeper analysis, but they ranked fifth and sixth in geNorm analysis and ninth and tenth in NormFinder analysis. For the total sample subset, BestKeeper analysis showed *CnRPL*, *CnUBC*, and *CnEF1α* as the top three stable genes, which were also the top three ranked genes in the geNorm and NormFinder analyses.

### 2.4. Comprehensive Ranking of the Candidate Reference Genes

As shown above, the evaluation results of gene expression stability varied when using different software such as geNorm, NormFinder, and BestKeeper (Table 2). To address this, we employed RankAggreg, an essential tool for integrating the stability rankings of candidate reference genes derived from various algorithms. This method utilizes a cross-entropy Monte Carlo approach or a genetic algorithm to generate aggregated, ordered lists based on the compiled rankings [62]. According to the integrated ranking results (Table 2), the three most stable genes for the abiotic stresses subset were *CnUBC*, *CnRPL*, and *CnEF1α*. For the hormonal stimulus subset, the three most stable genes were *CnRPL*, *CnEF1α*, and *CnGAPDH*. For the different tissue subset, *CnUBC*, *CnRPL*, and *CnCYP* were the top three stable genes. Across all samples, *CnRPL*, *CnUBC*, and *CnEF1α* were the most stable genes. Notably, *CnRPL* consistently ranked among the top three in stability across all subsets, suggesting that it could be a reliable reference gene for qRT-PCR studies in *C. nutans*.

### 2.5. Validation of the Recommended Candidate Reference Genes

**Figure 4:**
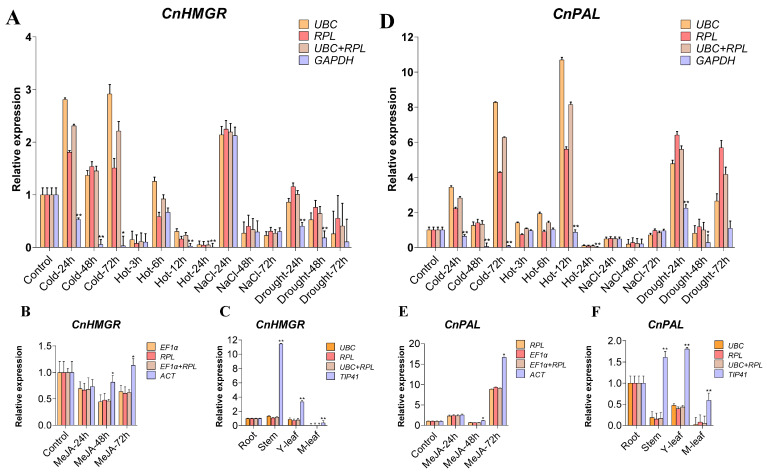
Relative expression patterns of *CnHMGR* and *CnPAL* in *C. nutans* were analyzed using qRT-PCR to validate the reliability of the recommended reference genes. Results were normalized against the most/least stable reference genes under abiotic stress (**A**,**D**) or hormone treatment (**B**,**E**), and in different tissues (**C**,**F**). Asterisks (* *p* < 0.05, ** *p* < 0.01) indicate significant differences in the expression levels of *CnHMGR* and *CnPAL*.

Despite the adverse effects of abiotic stresses on plant growth and development, they also enhance plant biochemical properties and the production of therapeutically beneficial secondary metabolites [63]. As research into the chemical composition and bioactivity of *C. nutans* advances, accumulating evidence indicates that triterpenoids and flavonoids are central to its pharmacological efficacy. In plants, the expression patterns of key genes are closely associated with the accumulation of secondary metabolites [64]. PAL plays a crucial role in the synthesis of phenolic compounds and flavonoids, and its activity level is directly related to developmental stages, tissues, organs, and plant genotypes. It also responds to environmental stress and plant hormones, with high expression leading to an increase in flavonoids [65]. HMGR is a key enzyme in the mevalonic acid pathway [66,67], and the isoenzymes of HMGR in different species undergo differential regulation under specific induction conditions, resulting in the accumulation of terpenoid metabolites [68,69,70]. To validate the reliability of the selected reference genes, we selected the most stable and least stable reference genes from each group to verify the relative expression levels of two key genes, *CnPAL* and *CnHMGR*, which are related to the accumulation of active substances in *C. nutans*, across different tissues and under various treatment conditions (Figure 4, Table S2).

Overall, when the two most stable reference genes were used as normalization factors, *CnHMGR* and *CnPAL* exhibited consistent trends in relative expression levels. However, inconsistent expression patterns were observed when the least stable reference genes were used for normalization. Specifically, under cold stress, using *CnUBC* (ranked 1) and *CnRPL* (ranked 2) as reference genes, both *CnHMGR* and *CnPAL* showed fluctuations in expression levels, characterized by an initial increase, followed by a decrease, and then another increase. By contrast, when the least stable gene (*CnGAPDH*) was used as a reference, the detected expression levels were significantly lower than those obtained with the top two reference genes, showing a completely opposite expression pattern. Under heat stress, fluctuations in *CnPAL* expression levels demonstrated a response to heat stress when the top two genes were used as reference genes, whereas no significant fluctuations were observed when the least stable gene was used. Across different tissue samples, using the two highest stability-ranked genes (*CnUBC*/*CnRPL*) as reference genes resulted in highly consistent expression trends for *CnHMGR* and *CnPAL*. Conversely, employing the least stable reference gene (*CnTIP41*) led to relative expression levels that were ten times higher than those obtained with the best two reference genes, indicating substantial fluctuations in *CnHMGR* and *CnPAL* expression profiles in stems and young leaves. This phenomenon was also evident in the MeJA-treated group. Our findings clearly indicated that using unsuitable reference genes as normalization factors in qRT-PCR analysis could yield inaccurate target gene expression results.

## 3. Discussion

*C. nutans* is a valuable traditional medicinal plant used in Southeast Asia and southern China. Both its leaves and roots can be used for medicinal purposes, containing various active compounds such as terpenes, steroids, flavonoids, and alkaloids. The synthesis of these secondary metabolites in plants is significantly influenced by various stress conditions and hormonal stimuli. Therefore, understanding the accumulation process of secondary metabolites in *C. nutans* requires exploring gene expression regulation under abiotic stresses and hormonal stimuli. Gene expression analysis is the initial and crucial step in elucidating the potential functions of target genes. qRT-PCR stands out as a powerful method for gene expression analysis, and the selection of suitable reference genes to normalize target gene expression is vital for minimizing experimental errors and ensuring reliable qRT-PCR results. Here, our study aimed to identify the most stable reference genes in *C. nutans* under different experimental conditions and in various tissues, thereby establishing a foundation for the accurate quantification of target genes using RT-qPCR.

**Figure 5:**
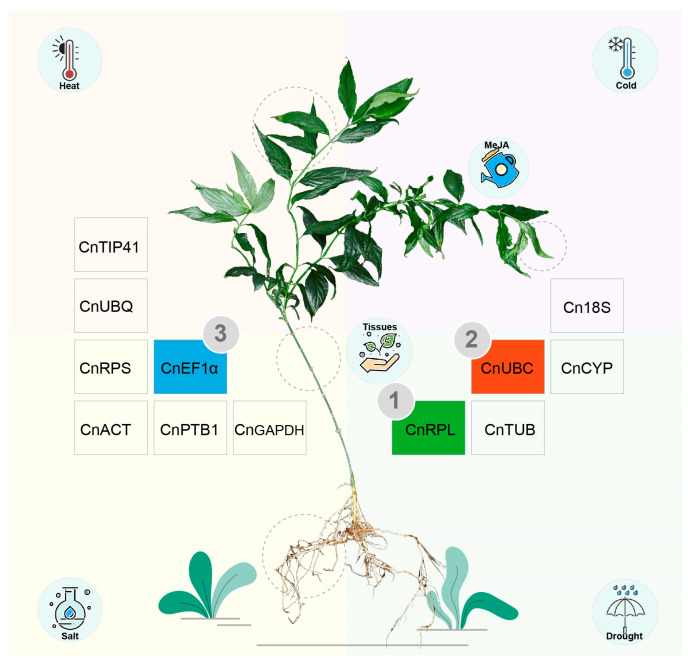
Summary of reference gene selection for *Clinacanthus nutans* across abiotic stresses, hormonal stimulus, and in different tissues. The central image shows a *C. nutans* plant, with gray dashed circles marking the tissue sampling locations. The circular icons represent the different experimental conditions. The numbers 1, 2, and 3 correspond to the top three reference genes according to the comprehensive ranking.

In this study, we selected 12 reference genes to evaluate their stability across different tissues and under various treatment conditions in *C. nutans*. The 12 candidate reference genes exhibited a broad expression range and high gene expression variation (Figure 2), indicating that no single gene consistently displayed constant expression under various conditions. To comprehensively analyze the most stable reference genes in *C. nutans*, we divided all test samples into four distinct subsets: “abiotic stress” (cold, heat, NaCl, and drought), “hormonal stimulus” (MeJA), “different tissues” (root, stem, young leaf, mature leaf), and “total” (all test samples). Then, three commonly used statistical algorithms, including geNorm, NormFinder, and BestKeeper, were employed to assess their stability. Under different abiotic stresses, all three methods identified *CnRPL*, *CnUBC*, *CnEF1α*, and *CnUBQ* as the most stable genes (Table 2). Similarly, in the total sample subset, these algorithms consistently ranked *CnRPL*, *CnUBC*, and *CnEF1α* as the top three most stable genes (Table 2), demonstrating their reliability across different statistical analyses. However, the results from these algorithms were not always congruent. For example, BestKeeper identified *CnUBC* as the most stable gene in the hormonal stimulus subset, whereas geNorm and NormFinder ranked it seventh and sixth, respectively. In the different tissues subset, BestKeeper identified *CnTUB* and *CnGAPDH* as the most stable genes, whereas geNorm and NormFinder ranked them fifth/sixth and ninth/tenth, respectively (Table 2). Similar phenomena have been reported in many other studies on the selection of reference genes in plants, with these discrepancies potentially stemming from the use of different algorithms [52,71]. Integrating the results from multiple software analyses can effectively mitigate such discrepancies [72,73]. According to the integrated ranking results (Table 2), the three most stable genes under abiotic stresses were *CnUBC*, *CnRPL*, and *CnEF1α*. Under hormonal stimulus, the three most stable genes were *CnRPL*, *CnEF1α*, and *CnGAPDH*. Across different tissue types, *CnUBC*, *CnRPL*, and *CnCYP* showed the highest stability. Overall, across all samples, *CnRPL*, *CnUBC*, and *CnEF1α* consistently emerged as the most stable genes. Notably, *CnRPL* consistently ranked among the top three stable genes across all subsets, possibly representing a relatively ideal choice for a reference gene in qRT-PCR analyses of *C. nutans* (Table 2, Figure 5). *RPL* genes encode ribosomal proteins that are integral components of the ribosome and are involved in the essential process of protein synthesis, which occurs in all cells [74]. Previous studies have shown that *RPL* genes exhibit remarkable stability across diverse plant species, rendering them reliable reference genes under various experimental conditions. For instance, *RPL1* has been identified as the most suitable reference gene in *Moringa oleifera*, demonstrating consistent stability across different developmental stages, tissues, and in response to various abiotic stress conditions [75]. In different tissue types and under various biotic stress conditions in apples, *RPL2* is considered one of the most suitable reference genes for different tissues and diverse stress conditions [76,77].

In previous studies, housekeeping genes such as *UBC*, *ACT*, *EF1α*, 1*8S*, *TUB*, and *GAPDH* were commonly selected as reference genes for qRT-PCR due to their perceived stable expression across all cells and physiological states, reflecting their critical roles in fundamental cellular structure and function. In our research, *CnUBC* also emerged as one of the top three stable genes across subsets involving different abiotic stress conditions, various tissue types, and all samples, collectively (Table 2). Meanwhile, *UBC* was also identified as the most suitable gene for various plant organs in *S. cusia* within the same family [28]. However, increasing evidence indicates significant variability in the expression levels of these traditional housekeeping genes depending on factors such as plant species, tissue, organ, and experimental conditions [27]. For instance, in our study, although *CnGAPDH* ranked among the top three stable genes in the different tissues subset, it exhibited the least stability under various stress conditions and across all sample subsets. Comparable instability of *GAPDH* has been documented in various peach samples and *Peucedanum praeruptorum* subjected to different experimental conditions [78,79]. Besides, *CnTUB* also demonstrated poor stability across different subsets of *C. nutans*, consistently ranking among the least stable genes in both the abiotic stress and hormonal stimulus subsets. Similarly, *GAPDH* and *TUB* in *S. cusia* also exhibit poor stability, suggesting a degree of similarity between plants within the same family. However, in *S. cusia*, *18S* stands out as the most stable gene under UV irradiation and hormonal stimulus conditions [28], while showing poor stability performance in *C. nutans* under different treatment conditions and in various tissues. Furthermore, in *A. paniculata*, *ACT* is identified as the most stable reference gene under MeJA treatment [44], whereas in *C. nutans*, it ranks last among the 12 candidate reference genes under the same treatment conditions (Table 2). Overall, absolute stability in gene expression is not universally observed in plants. Therefore, to ensure accurate results in qRT-PCR studies, it is critical to carefully select genes that demonstrate relatively stable expression under diverse experimental conditions as suitable reference genes for normalization.

In recent studies, novel reference genes like *TIP41*, *PTB1*, *SAND*, and *PP2A* have demonstrated robust stability across various tissues and under different experimental conditions. For instance, *TIP41* has been identified as the most stable reference gene in *Melaleuca bracteate* and *Salicornia europaea* under abiotic stresses [27,80]. It has also served as a reliable reference gene in different tissues of bamboo [81]. However, our research found *CnTIP41* to be among the least stable genes in the different tissues subset, abiotic stresses subset, and the total sample subset of *C. nutans*. *PTB1*, identified as one of the top three most stable genes in citrus under infection with *Candidatus Liberibacter asiaticus* conditions [82], exhibited less stability in all subsets in *C. nutans*, ranking between seventh and tenth place. These findings underscore the species-specific and condition-dependent nature of reference gene stability in plants.

Despite the systematic evaluation of reference gene stability in *C. nutans* under different experimental conditions and in various tissue types, certain limitations should be acknowledged. First, this study focused on a single variety of *C. nutans*, and gene expression stability may vary among different genotypes or cultivars. Thus, while our study examined the effects of temperature (heat, cold), salt stress, drought, and MeJA treatment, other environmental factors, such as light intensity fluctuations, heavy metal exposure, and soil nutrient availability, were not investigated. These factors may also influence reference gene stability and should be considered in future studies. Additionally, the reproducibility of qRT-PCR results can be affected by variations in experimental conditions and RNA quality, necessitating further validation across different laboratories and technical platforms. Future research should extend the evaluation to different cultivars, growth stages, and broader environmental variables to enhance the applicability of the selected reference genes. Moreover, integrating transcriptomic data or stability validation studies (e.g., RNA-seq combined with qRT-PCR) could provide more comprehensive insights into reference gene selection for *C. nutans* and related species.

## 4. Materials and Methods

### 4.1. Plant Materials and Stresses/MeJA Treatments

The *C. nutans* plants were sampled from the Fujian Agriculture and Forestry University, Fuzhou, Fujian Province. Stem cuttings, approximately 30 cm long, were taken from mature plants for propagation. After rooting and exhibiting vigorous growth, each cutting was transplanted individually into pots containing a mixture of nutrient soil and peat soil in a 2:1 ratio. The plants were grown in a greenhouse under controlled conditions of 28 °C, with a light/dark cycle of 16 h light and 8 h dark, and 75% humidity for 60 days. Robust and uniformly growing plants were selected for grouping experiments, with three biological replicates per group.

A total of four abiotic stress groups and one hormonal stimulus group were designed, comprising drought, salt, cold, heat, and methyl jasmonate (MeJA) treatment. Drought stress was simulated with 150 mmol/L mannitol solution, while salt stress was induced by 150 mmol/L NaCl solution. Cold stress was applied at 8 °C and heat stress at 45 °C. MeJA treatment was administered as a foliar spray with 200 μmol/L concentration until runoff. Young leaf samples from *C. nutans* materials were collected at 0, 24, 48, and 72 h after treatment (and at 0, 6, and 12 h for heat stress) alongside control samples. Additionally, samples from roots, stems, new leaves, and old leaves of normally growing *C. nutans* plants were collected to serve as the group representing different tissues. Thus, a total of 72 samples were analyzed, consisting of 60 stress-treated samples (young leaves subjected to cold, heat, drought, salt stress, and MeJA treatment) and 12 organ-specific samples (root, stem, young leaves, and mature leaves). All samples were immediately flash-frozen in liquid nitrogen and subsequently stored at −80 °C for future use.

### 4.2. RNA Isolation and cDNA Synthesis

Total RNA was extracted using TRIzol™ Reagent (Invitrogen, Thermo Fisher Scientific, Waltham, MA, USA) following the manufacturer’s protocol. Specifically, the processed *C. nutans* tissue was rapidly ground into a powder in liquid nitrogen. The powdered plant tissue was added to the centrifuge tube in a ratio of 1:2 with TRIzol (approximately 1 mL), vortexed for thorough mixing, and incubated for 5 min. Chloroform (200 μL) was then added to the tube, vortexed for 15 s and left to stand for 2 to 3 min before centrifugation at 12,000 r/min, 4 °C, for 15 min. The supernatant was transferred to an RNase-free centrifuge tube. An equal volume of isopropanol (approximately 500 μL) was added to the tube, gently mixed, incubated for 10 min, and centrifuged at 12,000 r/min, 4 °C, for 10 min. The supernatant was discarded, and the precipitate was resuspended in 1 mL of 75% ethanol, gently agitated, and then centrifuged at 7500 r/min, 4 °C for 5 min. The supernatant was discarded, and the centrifuge tube was allowed to air-dry on a clean bench for 5 to 10 min. Subsequently, 100 μL of RNase-free water was added to dissolve the precipitate, followed by storage at −80 °C. Total RNA integrity and concentration were assessed using 0.8% agarose gel electrophoresis and a Nanodrop2000 (Thermo Fisher Scientific, Waltham, MA, USA). cDNA first-strand synthesis was performed following the instruction of HiScript II Q RT SuperMix (Vazyme Biotech, Nanjing, China) for qPCR (+gDNA wiper), and the samples were stored at −20 °C for future use.

### 4.3. Selection of Potential Reference Genes and Primer Design

The selection of the 12 candidate reference genes was based on their extensive utilization in previous studies, particularly in gene expression normalization research involving other species within the family Acanthaceae. Specifically, we incorporated 10 candidate reference genes previously identified in *S. cusia* and 7 candidate reference genes reported in *A. paniculata*. Additionally, we included commonly used reference genes frequently employed in medicinal plant research, along with novel reference genes that have gained increasing attention in recent years. Finally, we ultimately identified 12 candidate reference genes, namely *CnTIP41*, *CnUBC*, *CnRPS*, *CnACT*, *CnPTB1*, *CnEF1α*, *Cn18S*, C*nCYP*, *CnTUB*, *CnUBQ*, *CnRPL*, and *CnGAPDH*. The gene sequences were obtained through de novo transcriptome assembly based on RNA-Seq data. The list of coding sequences (CDS) is provided in Table S3. Subsequently, following the principles of fluorescence-based quantitative PCR primer design, primers for the 12 candidate reference genes were designed using Primer 5.0 (Table S4), and synthesized by Fuzhou Shangya Biotechnology Co., LTD (Fuzhou, China).

### 4.4. Primer’s Specificity, Amplification Efficiency and qRT-PCR Analysis

The cDNA samples from all six experimental groups were mixed in equal amounts, diluted 5-fold, and utilized as templates for conventional PCR amplification. PCR products were subjected to 1% agarose gel electrophoresis to detect the specificity of the target bands. Equal amounts of the original cDNA samples from all experimental groups were mixed, then diluted to concentrations of 1/5, 1/25, 1/125, and 1/625, and analyzed by real-time PCR analysis. A standard curve was plotted using GraphPad software (V8.0.2) with the logarithm of the template dilution factor on the *x*-axis and the Ct value on the *y*-axis. The slope of the standard curve, denoted as K, was determined, and the amplification efficiency was calculated using the formula E = 5^−1/K^−1. qRT-PCR amplification was performed using the fluorescence-based quantification reagent, Taq Pro Universal SYBR qPCR Master Mix (Vazyme, Nanjing, China). The reaction mixture (20 μL) consisted of Taq Pro Universal SYBR qPCR Master Mix 10 (μL), forward and reverse primers (10 μmol/L) (0.4 μL each), cDNA (1 μL), and ddH_2_O (8.2 μL). The qPCR thermal cycling program included a two-step method: initial denaturation at 95 °C for 30 s, denaturation at 95 °C for 10 s, annealing at 60 °C for 30 s, and 40 cycles of denaturation and annealing. Following amplification, melting curve analysis was conducted with the following program: 95 °C for 15 s, 60 °C for 60 s, and 95 °C for 1 s.

### 4.5. Expression Stability Analysis of Candidate Reference Genes

The data from six experimental groups were examined, and raincloud plots depicting the distribution of Ct values for the 12 candidate reference genes across all samples were generated using R (v.4.3). Curves illustrating Ct value changes for each candidate gene under different treatments were plotted based on different treatment groupings. The average cycle threshold was calculated using data from three biological replicates. In accordance with previous reports, the average cycle threshold (Ct) value was calculated using data from three biological replicates to determine the expression level of each gene in the respective samples. The stability of gene expression was assessed using GeNorm, NormFinder, and BestKeeper software, and the endoGenes pipeline was employed for automated analysis of the three algorithms, available at https://github.com/hanielcedraz/endoGenes/tree/master (accessed on 12 August 2024). Specifically, GeNorm was utilized to generate stability M and pairwise variation V values, where a lower M value indicates more stable gene expression. Vn/n + 1 value < 0.15 indicates no additional reference genes are required for normalization. Finally, the R package RankAggreg was used for comprehensive evaluation and to determine the ranking order.

### 4.6. qRT-PCR Validation of Selected Reference Genes

To validate the reliability of the recommended reference genes, the expression patterns of two target genes, phenylalanine ammonia lyase (PAL) and HMG-CoA reductase (HMGR), were analyzed using qRT-PCR. PAL is a key enzyme in the phenylpropanoid pathway that is essential for the production of compounds such as anthocyanins, flavonoids, and phenylalanine. Plants also produce a variety of cyclic triterpenes, which are major products of the mevalonate (MVA) pathway, where HMGR serves as the rate-limiting enzyme. The qRT-PCR analysis was conducted as previously described, with three biological replicates. The primers for the target genes are listed in Table 1. The two most stable reference genes and one of the least stable reference genes were selected to standardize and normalize the expression of the target genes. Relative expression levels were determined using the comparative 2^−ΔΔCt^ method. The statistical tests of the gene expression data were performed using SPSS 22.0 software (SPSS Inc., Chicago, IL, USA).

## 5. Conclusions

Here, we conducted a systematic analysis of the stability of 12 candidate reference genes in *C. nutans* in various tissues (root, stem, young leaf, old leaf), under different abiotic stresses (cold, heat, NaCl, and drought), and after hormone treatment (MeJA). Integrated analysis revealed that *CnUBC*, *CnRPL*, and *CnEF1α* were the most suitable reference genes under abiotic stress conditions, while *CnRPL*, *CnEF1α*, and *CnGAPDH* exhibited the highest stability under hormone treatment. Across different tissues, *CnUBC*, *CnRPL*, and *CnCYP* were identified as the most stable genes, and overall, *CnRPL*, *CnUBC*, and *CnEF1α* were the most suitable reference genes under all tested conditions. To further validate the reliability of these reference genes, we analyzed the expression of two key biosynthetic genes, *CnPAL* and *CnHMGR*, using the most stable and least stable reference genes for normalization. The results showed that when the most stable reference genes were used, the expression patterns of the target genes were consistent with biological expectations. However, normalization with the least stable reference genes led to significant deviations in expression levels. These validation results were highly consistent with the evaluations from geNorm, NormFinder, and BestKeeper, further confirming the stability and applicability of our selected reference genes. These findings highlight the critical importance of selecting suitable reference genes as a prerequisite for qRT-PCR analysis.

## Figures and Tables

**Table 1:** Primer characteristics of 12 candidate reference genes.

Gene Name	Annealing Temperature (°C)	Amplicon Size (bp)	Primer Efficiency (%)	R^2^ Value
*CnUBQ*	60.1	70	92.02	0.9985
*CnRPL*	60	90	93.44	0.9983
*CnRPS*	60	125	96.1	0.9986
*CnPTB1*	59.8	145	98.35	0.9976
*CnTIP41*	60	101	95.83	0.9953
*CnACT*	59.9	178	102.4	0.9979
*CnUBC*	59.9	90	92.54	0.9981
*CnGAPDH*	60	91	93.86	0.9953
*Cn18S*	60	180	94.5	0.9925
*CnCYP*	60.2	141	97.97	0.9989
*CnEF1α*	60	132	92.12	0.998
*CnTUB*	60	160	89.78	0.9964

**Table 2:** Gene expression stability ranked by geNorm, NormFinder, BestKeeper, and Comprehensive ranking.

Group	Rank	geNorm		NormFinder		BestKeeper		Rank Aggreg
		Gene	MV Value	Gene	Stability	Gene	SD Value	
Abiotic stresses	1	*CnUBQ*	0.338135	*CnEF1α*	0.19	*CnRPL*	0.44	*CnUBC*
	2	*CnUBC*	0.338135	*CnUBC*	0.24	*CnUBC*	0.579	*CnRPL*
	3	*CnEF1α*	0.387752	*CnUBQ*	0.33	*CnEF1α*	0.678	*CnEF1α*
	4	*CnRPL*	0.474062	*CnRPL*	0.35	*CnUBQ*	0.701	*CnUBQ*
	5	*CnACT*	0.637643	*CnACT*	0.6	*CnTUB*	0.851	*CnACT*
	6	*CnRPS*	0.742216	*CnCYP*	0.62	*Cn18S*	0.945	*CnRPS*
	7	*Cn18S*	0.821862	*CnRPS*	0.73	*CnACT*	0.968	*Cn18S*
	8	*CnPTB1*	0.895786	*Cn18S*	0.8	*CnCYP*	0.994	*CnCYP*
	9	*CnCYP*	1.017078	*CnTIP41*	0.89	*CnRPS*	1.117	*CnPTB1*
	10	*CnTUB*	1.123396	*CnPTB1*	0.96	*CnPTB1*	1.148	*CnTUB*
	11	*CnTIP41*	1.215425	*CnTUB*	1.08	*CnTIP41*	1.149	*CnTIP41*
	12	*CnGAPDH*	1.365533	*CnGAPDH*	1.18	*CnGAPDH*	1.182	*CnGAPDH*
Hormonal stimulus	1	*CnRPL*	0.083542	*CnRPL*	0.09	*CnUBC*	0.32	*CnRPL*
	2	*CnEF1α*	0.083542	*CnGAPDH*	0.11	*Cn18S*	0.328	*CnEF1α*
	3	*CnGAPDH*	0.130138	*CnEF1α*	0.11	*CnRPL*	0.37	*CnGAPDH*
	4	*CnRPS*	0.161941	*CnRPS*	0.17	*CnUBQ*	0.399	*CnRPS*
	5	*CnTIP41*	0.197269	*CnTIP41*	0.21	*CnEF1α*	0.412	*CnUBC*
	6	*CnUBQ*	0.218152	*CnUBC*	0.22	*CnRPS*	0.425	*CnUBQ*
	7	*CnUBC*	0.234476	*CnCYP*	0.26	*CnPTB1*	0.434	*Cn18S*
	8	*Cn18S*	0.249184	*CnUBQ*	0.28	*CnGAPDH*	0.479	*CnTIP41*
	9	*CnCYP*	0.269206	*Cn18S*	0.33	*CnTIP41*	0.483	*CnCYP*
	10	*CnTUB*	0.344994	*CnTUB*	0.59	*CnCYP*	0.53	*CnPTB1*
	11	*CnPTB1*	0.434288	*CnPTB1*	0.67	*CnACT*	0.919	*CnTUB*
	12	*CnACT*	0.512254	*CnACT*	0.72	*CnTUB*	0.951	*CnACT*
Different tissues	1	*CnRPL*	0.220736	*CnUBC*	0.2	*CnTUB*	0.252	*CnUBC*
	2	*CnCYP*	0.220736	*CnEF1α*	0.34	*CnGAPDH*	0.31	*CnRPL*
	3	*CnUBC*	0.282248	*CnRPL*	0.46	*CnCYP*	0.381	*CnCYP*
	4	*CnEF1α*	0.346926	*CnUBQ*	0.57	*CnRPL*	0.395	*CnTUB*
	5	*CnTUB*	0.464707	*CnCYP*	0.57	*CnUBC*	0.564	*CnEF1α*
	6	*CnGAPDH*	0.519896	*Cn18S*	0.84	*CnPTB1*	0.596	*CnGAPDH*
	7	*CnPTB1*	0.617063	*CnPTB1*	0.91	*CnEF1α*	0.652	*CnPTB1*
	8	*Cn18S*	0.74218	*CnRPS*	0.92	*CnUBQ*	1.203	*CnUBQ*
	9	*CnUBQ*	0.818679	*CnTUB*	0.98	*Cn18S*	1.287	*Cn18S*
	10	*CnRPS*	0.918168	*CnGAPDH*	0.98	*CnRPS*	1.571	*CnRPS*
	11	*CnACT*	1.024491	*CnACT*	1.34	*CnACT*	1.863	*CnACT*
	12	*CnTIP41*	1.209021	*CnTIP41*	1.45	*CnTIP41*	1.97	*CnTIP41*
Total	1	*CnUBC*	0.404232	*CnEF1α*	0.2	*CnRPL*	0.444	*CnRPL*
	2	*CnEF1α*	0.404232	*CnUBC*	0.21	*CnUBC*	0.559	*CnUBC*
	3	*CnRPL*	0.464657	*CnRPL*	0.31	*CnEF1α*	0.648	*CnEF1α*
	4	*CnUBQ*	0.506615	*CnUBQ*	0.37	*CnTUB*	0.73	*CnUBQ*
	5	*Cn18S*	0.669644	*CnCYP*	0.57	*CnUBQ*	0.781	*Cn18S*
	6	*CnRPS*	0.768979	*CnRPS*	0.7	*CnCYP*	0.841	*CnCYP*
	7	*CnPTB1*	0.865782	*Cn18S*	0.72	*CnGAPDH*	0.923	*CnRPS*
	8	*CnACT*	0.949271	*CnACT*	0.74	*Cn18S*	0.938	*CnPTB1*
	9	*CnCYP*	1.034368	*CnTIP41*	0.81	*CnPTB1*	0.968	*CnTUB*
	10	*CnTUB*	1.121927	*CnPTB1*	0.9	*CnRPS*	1.145	*CnACT*
	11	*CnTIP41*	1.21541	*CnTUB*	0.98	*CnACT*	1.201	*CnTIP41*
	12	*CnGAPDH*	1.328414	*CnGAPDH*	1.02	*CnTIP41*	1.211	*CnGAPDH*

## Data Availability

The raw transcriptome sequencing data used in this study have been deposited in CNGBdb under the accession number CNP0006103. The datasets used and/or analyzed during the current study are available from the corresponding author upon reasonable request.

## Supplementary Materials

The following supporting information can be downloaded at: https://www.mdpi.com/article/10.3390/ijms26062483/s1.
