# Supplementary figures and images for: Selection and Validation of Reference Genes in Clinacanthus nutans Under Abiotic Stresses, MeJA Treatment, and in Different Tissues
Source: Int J Mol Sci. 2025 Mar 11;26(6):2483. doi: 10.3390/ijms26062483 (PMC11942611; doi:10.3390/ijms26062483)

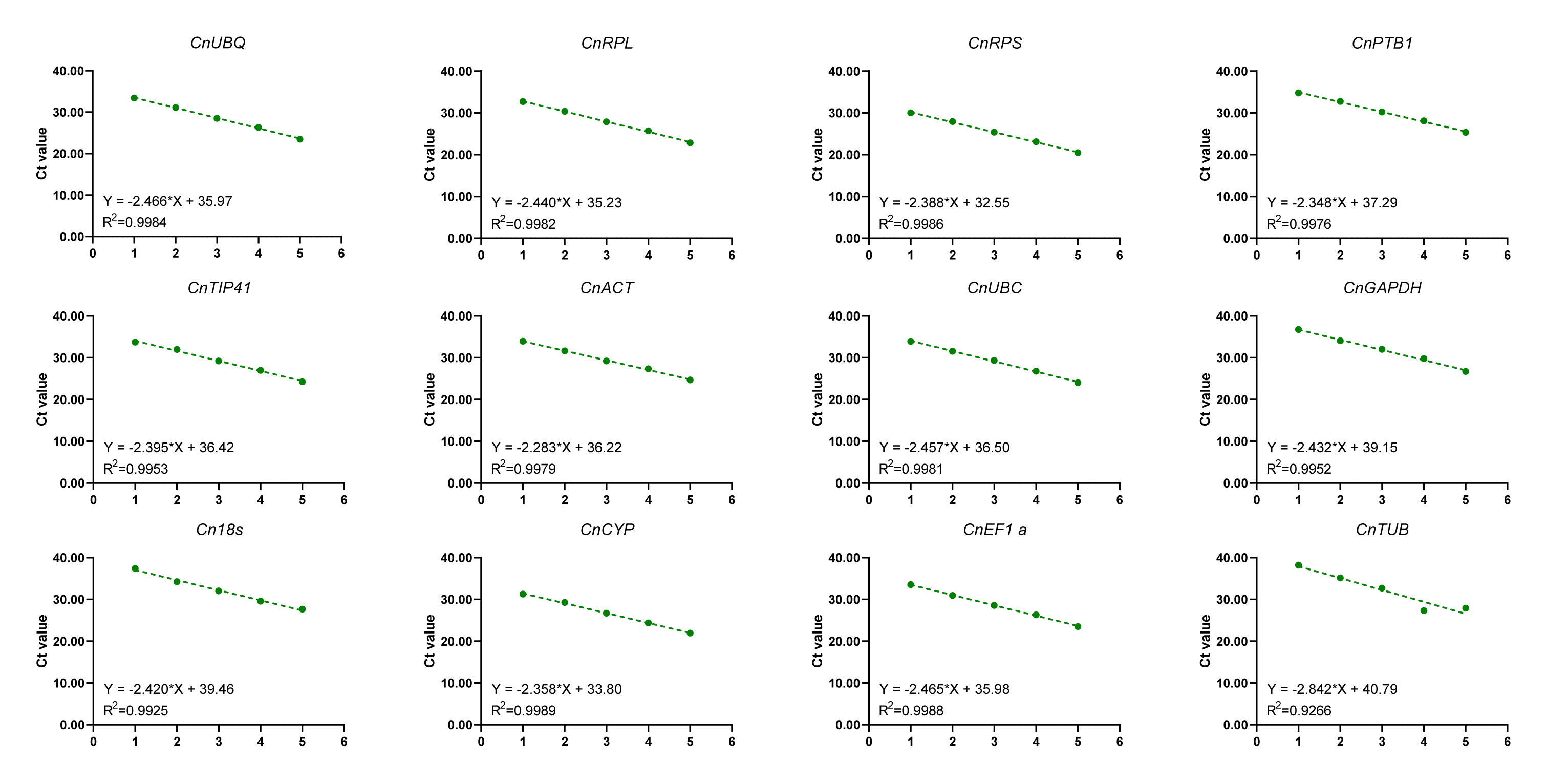

Supplement: Supplementary file 1 [file ijms-26-02483-s001.zip › 2.Supplementary_Materials/FigureS2.png]

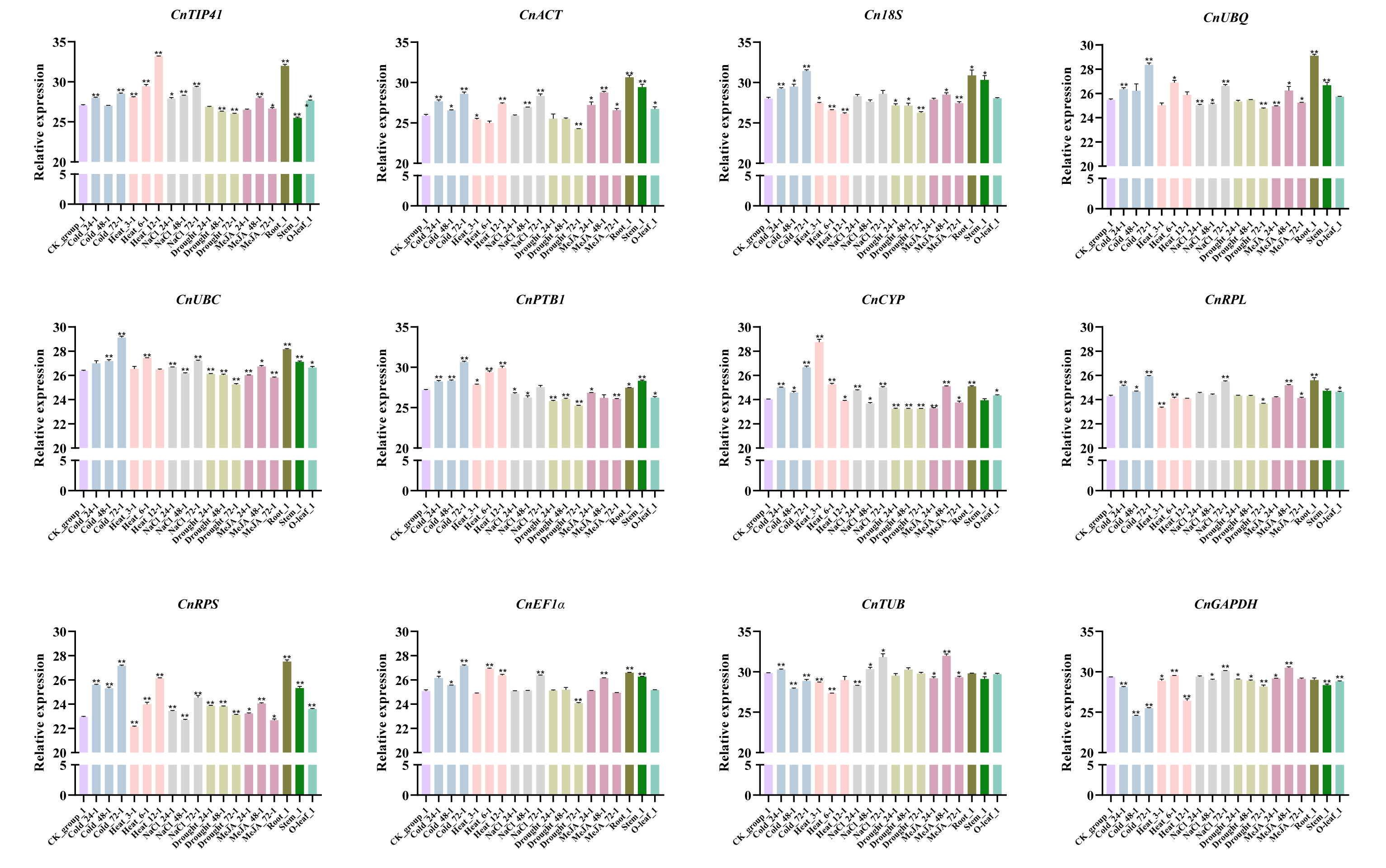

Supplement: Supplementary file 1 [file ijms-26-02483-s001.zip › 2.Supplementary_Materials/FigureS3.png]

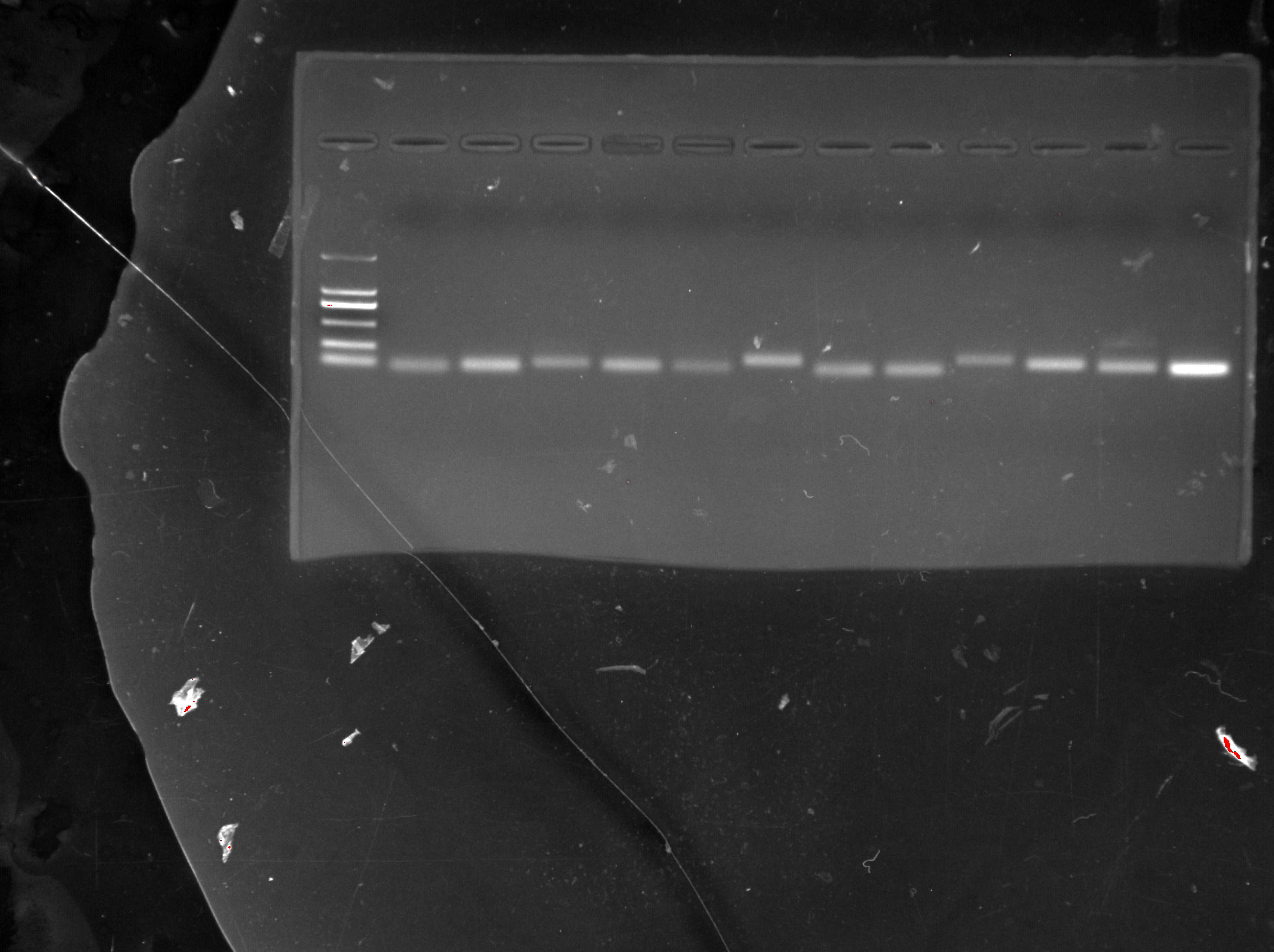

Supplement: Supplementary file 1 [file ijms-26-02483-s001.zip › 2.Supplementary_Materials/Figure_S1_original.tif]
